# A retrospective study: multiply recurrent intracranial germ cell tumors

**DOI:** 10.3389/fonc.2026.1962932

**Published:** 2026-10-19

**Authors:** Chuan Zhao, Jingjing Ge, Cheng Li, Chi Zhao, Shaopei Qi, Fengjun Xue, Jingjing Zhang, Junping Zhang

**Affiliations:** 1 Department of Neurosurgery, Sanbo Brain Hospital, Capital Medical University, Beijing, China; 2 Department of Neuro-oncology, Sanbo Brain Hospital, Capital Medical University, Beijing, China

**Keywords:** dissemination, intracranial germ cell tumors, management, prognosis, recurrence

## Abstract

**Purpose:**

Intracranial germ cell tumors (GCTs) are a rare group of malignancies that are prone to recurrence and dissemination. The management of patients with multiple recurrent GCTs is particularly challenging. This study aimed to summarize the clinical characteristics of intracranial GCTs with multiple recurrences (≥3 times) and to provide a basis for improving prognosis.

**Methods:**

We collected clinical data from patients with multiply recurrent (≥3 times) intracranial GCTs treated at Sanbo Brain Hospital between 2016 and 2023. Data included sex, age, histological diagnosis, number of recurrences, tumor location, treatment regimens, and survival outcomes. Treatment modalities at initial diagnosis and each recurrence were recorded. Progression-free survival (PFS) intervals between recurrences were compared, and overall survival (OS) was analyzed.

**Results:**

A total of 49 patients were included: 13 with pure germinomas and 36 with non-germinomatous germ cell tumors (NGGCTs). The cohort included 39 (79.6%) males and 10 (20.4%) females, with a mean age at initial presentation of 13.29 ± 5.99 years. The number of recurrences ranged from 3 to 11. Overall survival of germinoma patients was significantly better than that of NGGCT patients (P = 0.0019). For both germinomas and NGGCTs, the PFS interval between first and second recurrences (PFS2) was significantly shorter than that from initial diagnosis to first recurrence (PFS1) (P = 0.0286 and P = 0.0428, respectively). The Friedman test demonstrated significant shortening of PFS intervals with successive recurrences in both germinomas (P<0.0001) and NGGCTs (P = 0.0092).

**Conclusion:**

For patients with multiply recurrent GCTs, management increasingly relies on systemic chemotherapy as local modalities become less feasible. In addition, the interval between recurrences shortens with successive relapses, with the time to the second recurrence being significantly shorter than that to the first in both germinomas and NGGCTs.

## Highlights

- Recurrence intervals shorten significantly with each relapse
- Multiply recurrent GCTs shift toward systemic chemotherapy
- NGGCT has worse overall survival than germinoma

## 1 Introduction

Identifying the precise clinical characteristics, treatment patterns, and outcomes of intracranial germ cell tumors with multiple recurrences is crucial to improving the management of this complex disease and guiding future research. Germinomas are highly sensitive to radiotherapy and chemotherapy and generally have an excellent prognosis. On the other hand, non-germinomatous germ cell tumors (NGGCTs) have variable prognoses due to their distinct pathological characteristics and treatment approaches (1–8).

Regardless of the tumor type, repeated recurrence means the patient must endure more suffering, and society bears a tremendous burden (9–14). Identifying the precise clinical characteristics of intracranial germ cell tumors with multiple recurrences is crucial to improving the management and outcomes of this complex disease (15).

Previous studies have reported that the overall survival rate for intracranial germ cell tumors is generally favorable, with 5-year survival rates ranging from 70% to 90% (3, 16). However, these studies have primarily focused on initial treatment and outcomes, with limited data on patients with multiple recurrences. A comprehensive understanding the clinical features, treatment strategies, and prognosis of intracranial multiply recurrent GCTs with is essential to optimizing patient care and guiding future research.

Therefore, this study aimed to comprehensively investigate the clinical characteristics, treatment approaches, and outcomes of patients with multiply recurrent intracranial GCTs. By analyzing this real-world data, we aimed to provide insights into the management of this difficult-to-treat population and contribute to inform clinical practice for patients with recurrent intracranial GCTs.

## 2 Materials and methods

### 2.1 Patients

We retrospectively collected the clinical data from patients with intracranial germ cell tumors who were treated at the Department of Neuro -oncology, Sanbo Brain Hospital between 2016 and 2023.This study was approved by the Institutional Review Board of Sanbo Brain Hospital, and the requirement for informed consent was waived due to the retrospective nature of the analysis. The inclusion criteria for this study were: (1) a confirmed diagnosis of intracranial germ cell tumor, and (2) a history of multiple recurrences (defined as three or more recurrences). The baseline characteristics of the included patients are summarized in **Table 1**.

**Table 1:** Characteristics of patients.

Factors		Number
Sex	Male	39 (79.6%)
	Female	10 (20.4%)
Age (initial diagnosis)	(mean)	13.29 ± 5.99 years
Diagnosis	Germinoma	13 (26.5%)
	NGGCT	36 (73.5%)
Times of recurrences	3	30(61.2%)
	4	10 (20.4%)
	5	4 (8.2%)
	6 and above	5(55.6%)
Positions (initial diagnosis)	Only pineal region	6(15.4%)
	Only sellar region	3(7.69%)
	Pineal and sellar	4(10.25%)
	Involving basal ganglia	4(10.25%)
	Other locations	22(56.41%)

The data collected for each patient included: (1) Demographic information: sex, age; (2) Tumor characteristics: histological subtype, location of initial and recurrent tumors; (3) Treatment details: surgical interventions, radiation therapy, chemotherapy regimens, and any associated adverse events; (4) Outcomes: progression-free survival, overall survival, and status at the last follow-up.

Recurrence was defined as the reappearance or progression of tumor after initial response, confirmed by at least one of the following: (1) new or enlarged lesions on contrast-enhanced MRI, assessed by the treating neuroradiologist; (2) histological confirmation of viable tumor; or (3) a sustained rise in serum AFP or HCG above the upper limit of normal. All recurrences were reviewed and confirmed by the treating neuro-oncology team.

We defined PFS1 as the interval from initial diagnosis to first recurrence; PFS2 as the interval from first to second recurrence; and PFS3 as the interval from second to third recurrence.

### 2.2 Statistical analysis

All statistical analyses were performed using GraphPad Prism version 8 and R software (v 4.3.1). Survival curves were estimated using the Kaplan-Meier method, and differences between groups were assessed using the log-rank test. Univariate and multivariate Cox proportional hazards regression models were employed to identify factors associated with PFS before the first recurrence. A two-sided *P* < 0.05 was considered statistically significant for all analyses.

## 3 Results

### 3.1 Patient characteristics

The baseline characteristics of the study cohort are summarized in **Table 1**. A total of 49 patients with multiply recurrent intracranial GCTs (≥3 recurrences)were identified. The cohort consisted of 39 male (79.6%) and 10 female (20.4%) patients, with a mean age at symptom onset of 13.29 ± 5.99 years. Histologically, 13 patients (26.5%) had pure germinoma, while 36 patients (73.5%) had non-germinomatous germ cell tumors (NGGCTs). The distribution of patients based on the number of recurrences was as follows: 3 recurrences (n = 30, 61.2%),4 recurrences (n=10, 20.4%),5 recurrences (n=4, 8.2%),6 recurrences (n=2, 4.1%), 7 recurrences (n=1, 2.0%),8 recurrences (n=1, 2.0%),11 recurrences (n=1, 2.0%).

### 3.2 Germinomas

#### 3.2.1 Characteristics and treatment of recurrent germinomas

A total of 13 patients were diagnosed with germinomas, comprising 9 males and 4 females. The distribution of the number of recurrences in this subgroup was as follows: 3 recurrences: 9 patients (69.2%), 4 recurrences: 2 patients (15.4%), 5 recurrences: 2 patients (15.4%) (**Table 2**).

**Table 2:** Treatment details at each disease stage for multiply recurrent germinomas.

Disease stage	Patients treated (n)	Surgery (n)	Radiotherapy (n)	Chemotherapy (n)	Other therapies (n)	Notes
Initial Diagnosis	13	Biopsy: 7	13	5	–	No disseminated disease at diagnosis. All patients received RT as part of primary treatment.
First Recurrence	13	3	7	11	–	All patients received further treatment.
Second Recurrence	13	1	6	12	–	All patients received additional treatment.
Third Recurrence	11	4	4	11	Immunotherapy: 1	2 patients did not receive further treatment.
Fourth Recurrence	3	0	0	3	–	4 patients reached this stage; 1 received no further treatment.

RT, radiotherapy.

#### 3.2.2 Progression-free survival

The median progression-free survival from initial diagnosis to the first recurrence was 25.03 months (range 6.30-102.20 months). The median PFS from the first to the second recurrence was 14.16 months (range 2.14 - 72.01 months), and the median PFS from the second to the third recurrence was 13.50 months (range 5.03 - 108.05 months).

The median PFS2 (14.16 months) was significantly shorter than the median PFS1 (25.03 months) (*P* = 0.0286, **Figure 1A**), whereas no significant differences were observed while between PFS2 and PFS3 (*P* = 0.8263)or PFS1 and PFS3 (*P* = 0.1457).

**Figure 1:**
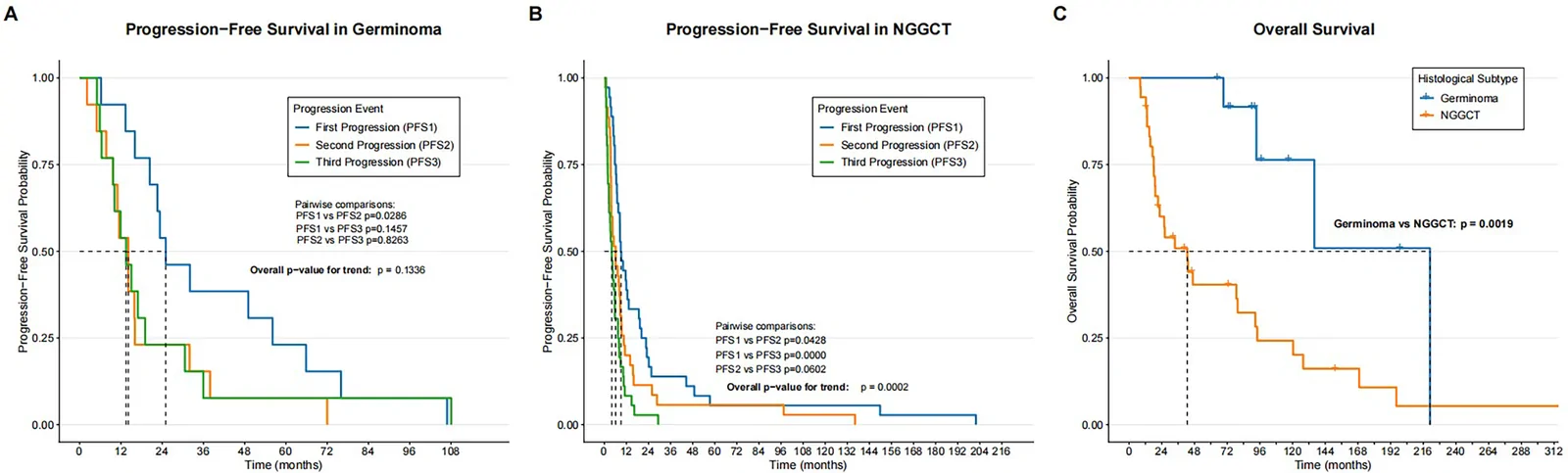
Comparison of survival curves. **(A)** In germinomas, the interval between the second (PFS2) was significantly shorter than that between onset and first recurrence (PFS1) (*P* = 0.0286). **(B)** In NGGCTs, PFS2 was also significantly shorter than PFS1 (*P* = 0.0428). **(C)** Comparison of overall survival between germinoma and NGGCT.

The Friedman test revealed a statistically significant decreasing trend in progression-free survival intervals with successive recurrences for germinomas (*P* < 0.0001).

#### 3.2.3 Overall survival

The overall survival of germinoma patients was significantly different from that of NGGCT patients (*P* = 0.0019, **Figure 1C**).

#### 3.2.4 Multivariate analysis

1. Progression-free Survival at first Recurrence

A multivariate analysis was performed to identify factors associated with progression-free survival (PFS) from the initial diagnosis to the first recurrence. The analysis of PFS of the first recurrence showed no statistical significance for age at diagnosis, sex, tumor location, presence of dissemination, radiotherapy, chemotherapy, resection, biomarkers (**Figure 2**).

**Figure 2:**
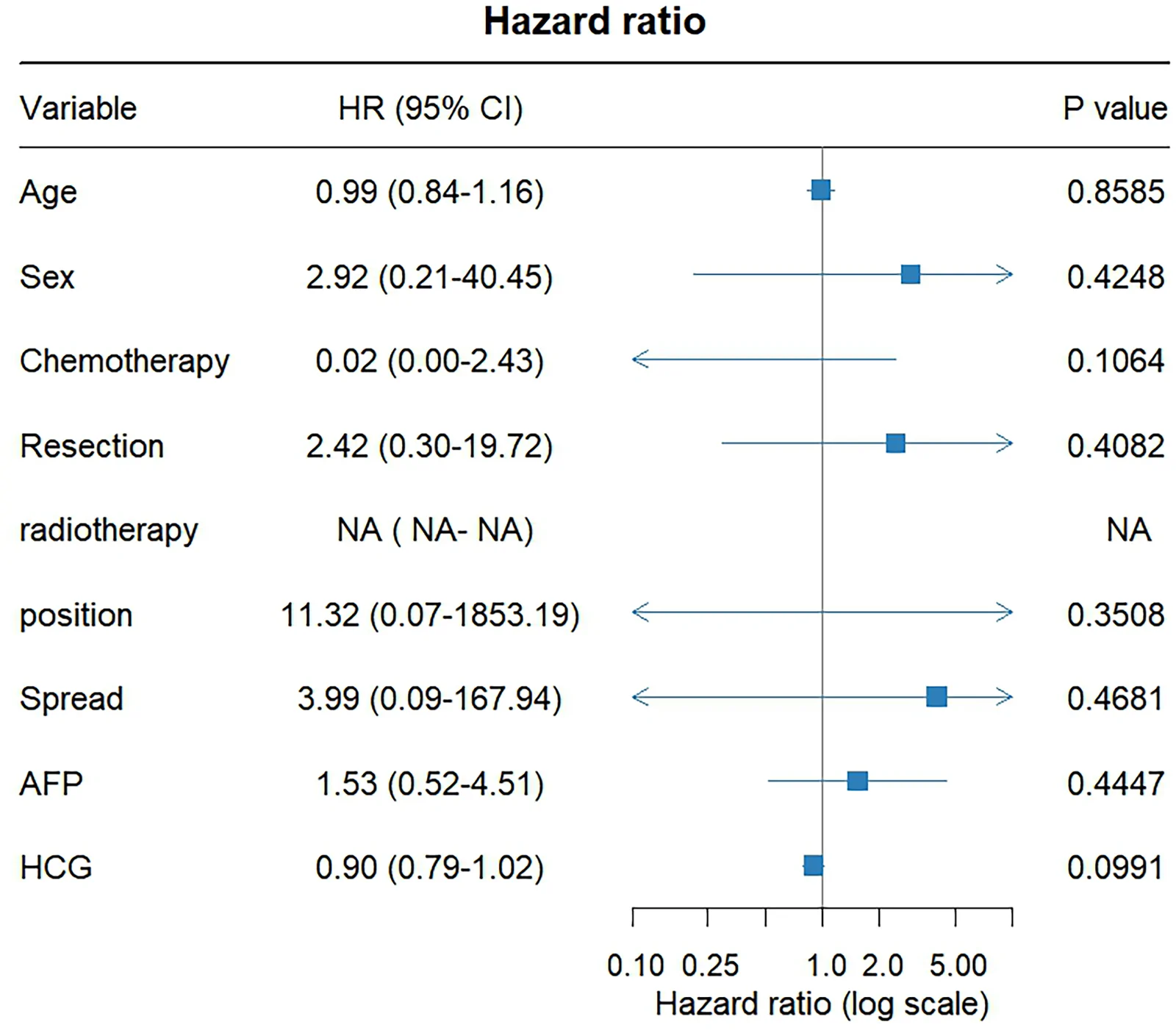
Multivariate analysis of PFS of the first recurrence of germinomas showed no statistical significance for age at diagnosis, gender, tumor location, presence of dissemination, radiotherapy, chemotherapy, resection, spread, biomarker. AFP, alpha-fetoprotein; HCG, human chorionic gonadotropin (elevated thresholds: AFP > 10 ng/mL, HCG > 5 IU/L for germinoma; thresholds for NGGCT were according to institutional standards). ‘Position’ refers to tumor location (suprasellar, pineal, bifocal, other). ‘Spread’ refers to the presence of disseminated disease at diagnosis.

2. Progression-free Survival at second Recurrence

Similarly, a multivariate analysis of PFS from the first to the second recurrence did not reveal any statistically significant predictors.

3. Overall Survival

A multivariate analysis of OS also did not identify any statistically significant prognostic factors in this cohort of patients with multiple recurrences of germ cell tumors.

### 3.3 Multiple recurrences in NGGCT

#### 3.3.1 Characteristics and treatment of recurrent NGGCTs

NGGCT comprised 36 patients, including 30 males and 6 females. The gender distribution was not significantly different from that of germinoma (*P* = 0.4223). The number of recurrences was 3 in 21 cases, 4 in 8 cases, 5 in 2 cases, 6 in 2 cases, 7 in 1 case, 8 in 1 case, and 11 in 1 case (**Table 3**). The number of recurrences in NGGCT was not statistically different from that in germ cell tumors, with a median of 3 (*P* = 0.4610, **Figure 3**).

**Table 3:** Treatment details at each disease stage for multiply recurrent NGGCTs.

Disease stage	Patients treated (n)	Surgery (n)	Radiotherapy (n)	Chemotherapy (n)	Other therapies (n)	Notes
Initial Diagnosis	36	22	25	25	–	4 patients had disseminated disease at diagnosis.
First Recurrence	36	11	15	26	–	All patients received treatment.
Second Recurrence	36	8	9	29	Targeted Therapy: 1	All patients received treatment.
Third Recurrence	32	5	6	26	Targeted Therapy: 1	4 patients had no clear further treatment.

NGGCT, non-germinomatous germ cell tumor.

**Figure 3:**
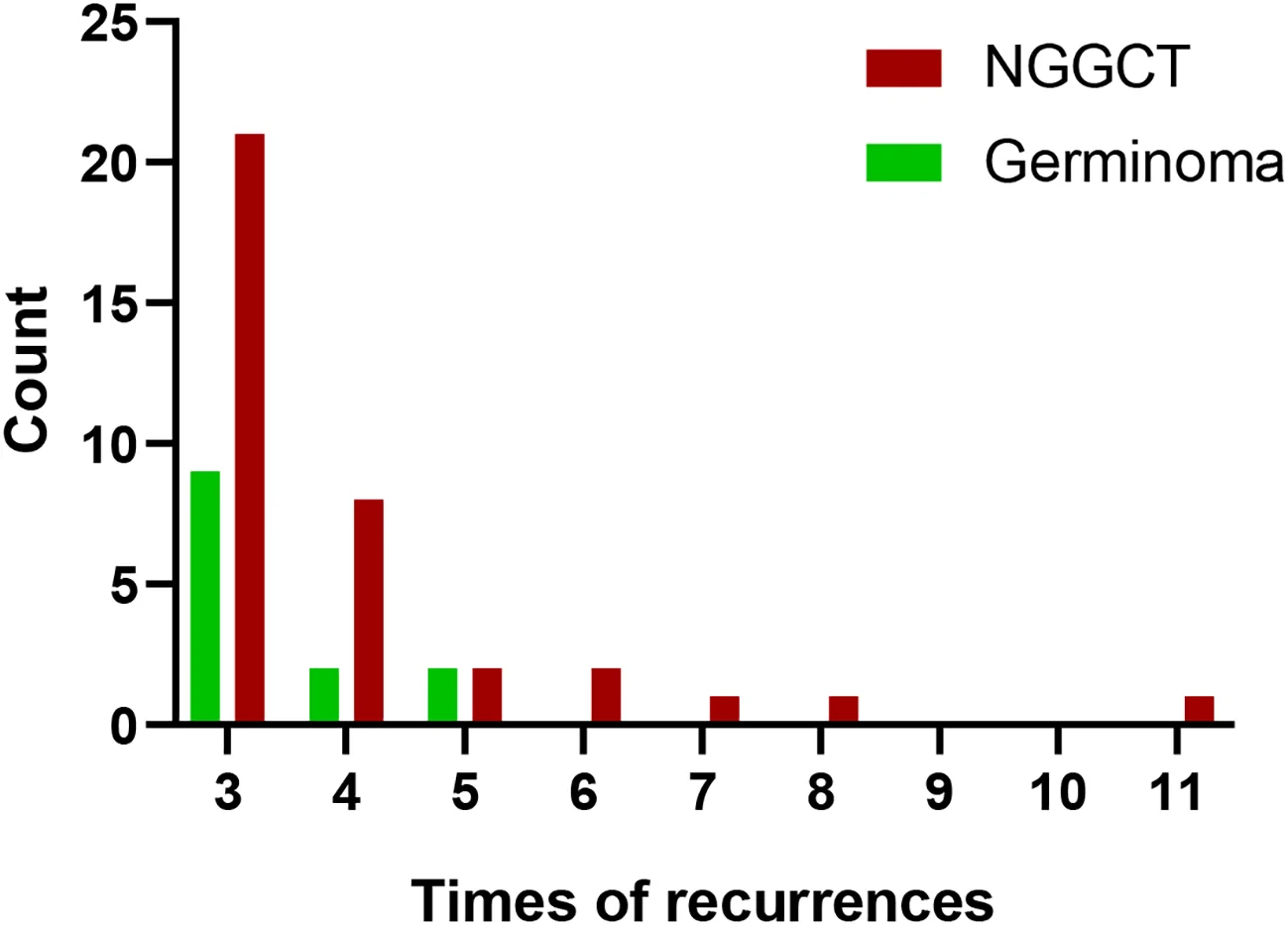
Comparison of recurrence times between germinoma and NGGCT showed no statistical difference (*P* = 0.4610).

#### 3.3.2 Progression-free survival

The median progression-free survival (PFS) from diagnosis to the 1st recurrence was 9.05 months (range: 0.97-201.87 months); from the 1st to the 2nd recurrence, it was 5.61 months (range: 0-136.17 months); and from the 2nd to the 3rd recurrence, it was 4.02 months (range: 0.60-29.20 months). Median PFS2 and PFS3 were both significantly shorter than median PFS1 (5.61 vs. 9.05 months, *P* = 0.0428; 4.02 vs. 9.05 months, *P* < 0.0001; **Figure 1B**). However, no significant difference was observed between PFS2 and PFS3 (5.61 vs. 4.02 months, *P* = 0.0602).

The Friedman test revealed a statistically significant decreasing trend in progression-free survival intervals with successive recurrences for germinomas (*P* = 0.0092).

#### 3.3.3 Multivariate analysis

Multivariate analysis of PFS of the first recurrence showed no statistical significance for age at diagnosis, sex, tumor location, presence of dissemination, radiotherapy, or resection. However, chemotherapy at initial diagnosis was independently associated with shorter PFS1 in NGGCTs (*P* < 0.001) (**Figure 4**). Additionally, similar statistical significance was found in the multivariate analysis of PFS after the second recurrence or overall survival.

**Figure 4:**
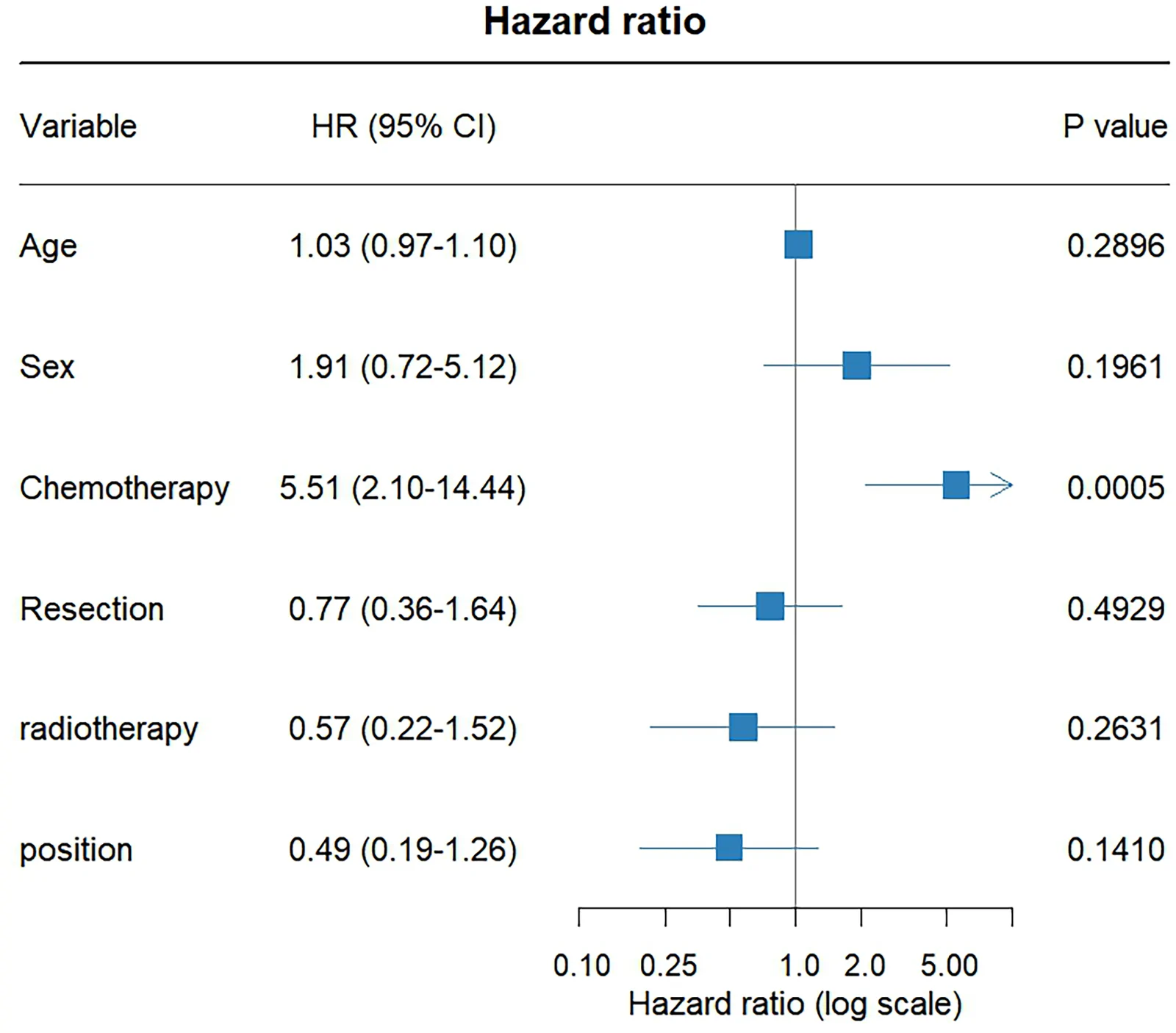
Multivariate analysis of PFS of the first recurrence of NGGCTs showed no statistical significance for age at diagnosis, sex, tumor location, presence of dissemination, radiotherapy, or resection.

#### 3.3.4 Comparison between patients with 3 recurrences and those with ≥4 recurrences

We compared patients with exactly 3 recurrences (n = 30) and those with ≥4 recurrences (n = 19). No significant differences were found in sex (male: 83.3% vs 78.9%; *P* = 0.2331), age at symptom onset (median, 12 vs 14 years; *P* = 0.7893), or diagnosis (germinoma: 30.0% vs 21.1%; *P* = 0.7408). Median PFS was 16.00 vs 11.90 months (log-rank *P* = 0.1416); median OS was not reached in either group (log-rank *P* = 0.3753).

In NGGCT (n = 36), median AFP was 357.95 vs 529.80 μg/L (*P* = 0.2066) and median HCG was 26.55 vs 51.53 IU/L (*P* = 0.1379). In pure germinoma (n = 13), median AFP was 2.00 vs 1.21 μg/L (*P* = 0.0956) and median HCG was 9.50 vs 1.52 IU/L (*P* = 0.5818). None reached statistical significance.

### 3.4 The most recurrent patients

Case 1. A 9-year-old girl presented in September 2013 with headache, nausea, vomiting, limb weakness, and right ptosis. MRI revealed a suprasellar mass (AFP 0.85 μg/L, HCG 0.9 IU/L). She underwent craniotomy on September 22, 2013, and pathology confirmed a germ cell tumor. She received whole-brain radiotherapy (34 Gy) with a sellar boost (48 Gy/20 fractions) and six cycles of BEP (cisplatin, etoposide, bleomycin), complicated by grade 4 leukopenia and grade 3 thrombocytopenia.

She subsequently experienced five recurrences: intraspinal dissemination (August 2015), spinal dissemination at L1–S1 (October 2016), intracranial dissemination with a rise in HCG to 462 IU/L (December 2017), ventricular and suprasellar dissemination (July 2018), and progressive intracranial disease (September 2018). Treatments included repeated BEP, spinal radiotherapy, ifosfamide–carboplatin–etoposide, pemetrexed–cisplatin–Endostar, whole-brain plus local radiotherapy, and temozolomide plus anlotinib. She died in October 2021 from disease progression; overall survival from diagnosis was 8 years and 1 month (**Figure 5**).

**Figure 5:**
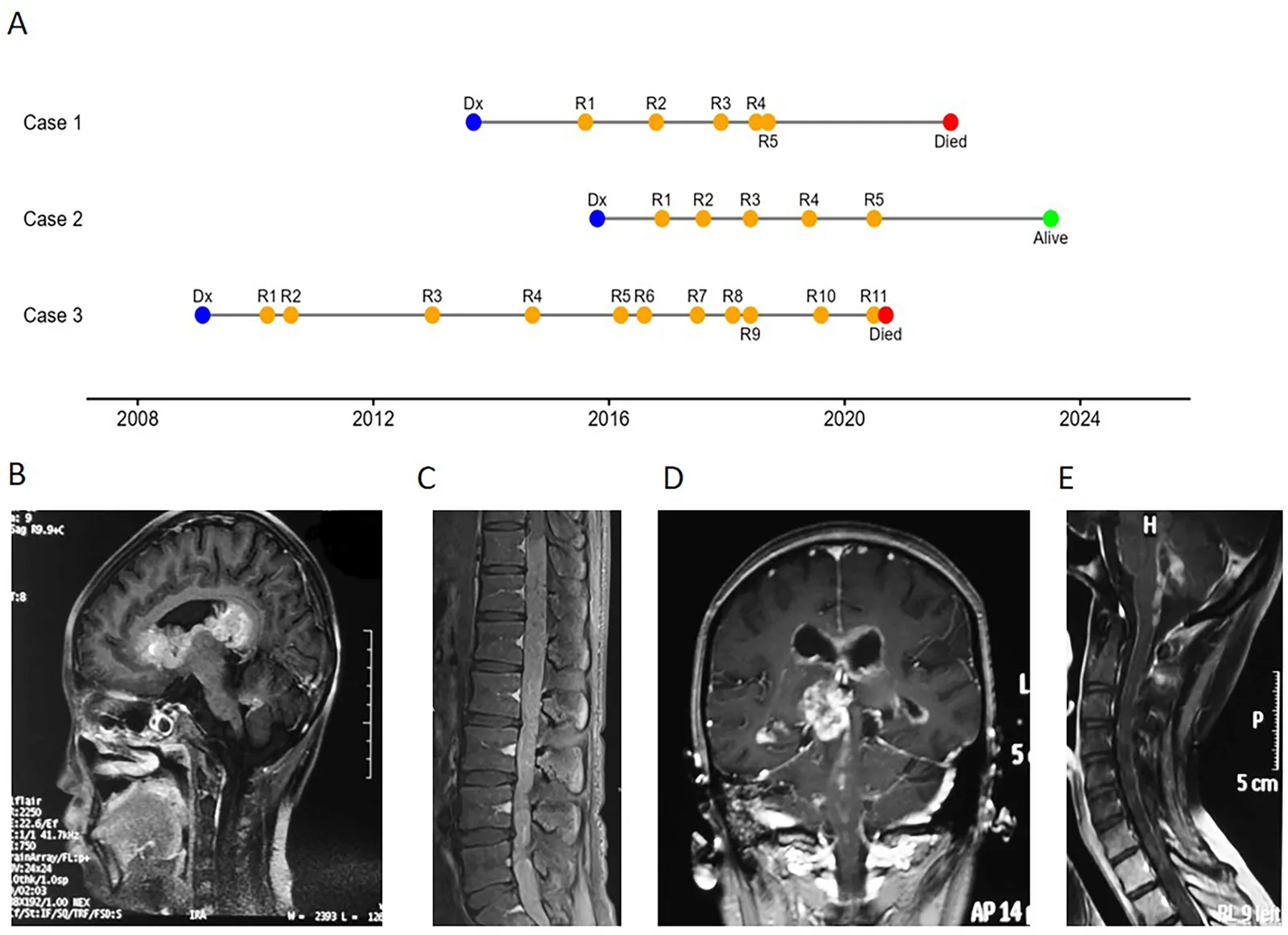
Timelines and representative neuroimaging of three patients. **(A)** Timelines of Cases 1–3. **(B–E)** Representative MRI: **(B)** Case 1, Dissemination involving the lateral and fourth ventricles; **(C)** Case 2, Intraspinal dissemination at the first recurrence; **(D)** Case 3, pineal lesions and eleventh recurrence with pineal, ventricular, and meningeal dissemination. **(E)** Case 3, fourth ventricular, cervical spinal, and intraspinal dissemination at the eleventh recurrence. Blue, diagnosis; orange, recurrence; red, death; green, alive.

Case 2. A 16-year-old boy presented in October 2015 with a 1-month history of headache, nausea, and vomiting. Brain MRI revealed a pineal mass (AFP 1.13 μg/L, HCG 2.15 IU/L), consistent with a germ cell tumor. He received whole-ventricular radiotherapy with a local boost to 50 Gy, achieving complete remission. He subsequently experienced five recurrences: extensive intraspinal lesions below T11 (November 2016), anterior cranial fossa and fourth ventricular lesions (July 2017), ventricular and brainstem dissemination (May 2018), multiple intracranial lesions (May 2019), and cerebellar, brainstem, and upper cervical dissemination (June 2020). Treatments included BEP, IFO–DDP–VP-16, spinal radiotherapy (30.06 Gy/18 fractions, boosted to 36.74 Gy/22 fractions), IFO–CBP–VP-16, and whole-ventricular plus focal radiotherapy (30.6 Gy/17 fractions). A biopsy in June 2020 confirmed germinoma involvement of the brain parenchyma. Grade 3–4 hematological toxicities occurred during chemotherapy. At the last follow-up in June 2023, he was alive without recurrence but had impaired balance and memory (**Figure 5**).

Case3.NGGCT case:

A 14-year-old boy presented in January 2008 with polydipsia and polyuria; brain MRI in August 2008 revealed a pituitary mass. In February 2009, he developed blurred vision and bitemporal hemianopsia (AFP 4.18 μg/L, HCG 221 IU/L), and repeat MRI showed sellar and pineal lesions. The sellar tumor was resected via an orbital keyhole approach, and pathology showed germinoma with mature teratoma components. He received local (30 Gy/18 fractions) and whole-brain (30 Gy/17 fractions) radiotherapy from March 2009.He subsequently experienced eleven recurrences: C5–7 intraspinal recurrence (March 2010); right tentorium and craniocervical junction lesions (August 2010); suprasellar, corpus callosum, and left frontal dissemination (January 2013); left ventricular spread (September 2014); left ventricular and right occipital lesions (March 2016); L2 spinal canal, right cerebellar angle, and right ventricular angle dissemination (August 2016); suprasellar and retroventricular recurrence (July 2017); right ventricular spread (February 2018); multiple disseminated lesions (May 2018); multiple ventricular and suprasellar lesions with markedly elevated markers (AFP 888.5 μg/L, HCG 148 IU/L; August 2019); and pineal, ventricular, and meningeal dissemination (July 2020).Treatments included surgery, whole-spinal and local radiotherapy, gamma knife radiosurgery, BLM–DDP–VM-26, IFO–DDP–VM-26, pemetrexed–cisplatin, apatinib, and IFO–CBP–VP-16 plus Endostar. Grade 3–4 hematological toxicities occurred repeatedly. He eventually died from disease progression (**Figure 5**).

## 4 Discussion

Intracranial germ cell tumors account for a small proportion of all intracranial tumors, but they are highly malignant (1, 3, 4, 6–8). Among them, germinomas are sensitive to radiotherapy and chemotherapy, with high cure rates and low recurrence rates. In contrast, NGGCTs have a more heterogeneous and often less favorable prognosis due to their diverse histology (e.g., teratoma, yolk sac tumor, choriocarcinoma) and variable responses to treatment (2, 3).

To clarify the specific situation of repeated recurrence of GCTs, we conducted this study. For pure germinomas, previous studies have shown that they account for about 2/3 of intracranial GCTs. The recurrence rate after standard radiotherapy and chemotherapy is very low. However, in our cohort, germinomas accounted for 13 of the 49 patients (26.5%) with multiply recurrent GCTs. This proportion is still considerable and is consistent with previous reports suggesting that a subset of germinomas exhibit an aggressive, recurrent course (2). The presence of this aggressive, multiply recurrent germinoma subgroup in our cohort suggests that a subset of tumors may be intrinsically resistant to conventional radiotherapy and chemotherapy. Therefore, it is reasonable to speculate that alternative therapeutic strategies, such as immunotherapy or targeted therapy, could be explored for these refractory cases, guided by their specific molecular characteristics (17–19).

Multivariate analysis of progression-free survival after the first recurrence showed no statistically significant differences in age at diagnosis, sex, tumor location, dissemination, radiotherapy, chemotherapy, or surgery. Nevertheless, the direction of the hazard ratios suggested a trend toward longer PFS in patients who received chemotherapy and toward shorter PFS in those with more extensive tumor involvement or dissemination. These findings should be interpreted as hypothesis-generating rather than definitive, given the limited statistical power of our cohort. In our cohort, chemotherapy at initial diagnosis emerged as a risk factor for shorter PFS1 in NGGCTs on multivariate analysis. It is critical to note that this analysis evaluated variables in isolation. In clinical practice, chemotherapy for NGGCT is rarely used alone but rather in conjunction with radiotherapy. In our data, the majority of patients (69.4%, 25/36) underwent multimodal therapy (chemotherapy with radiotherapy). Therefore, this result likely reflects the selection of more intensive chemotherapy for patients with higher-risk disease features at presentation, rather than indicating a detrimental effect of chemotherapy per se. This underscores the importance of risk-adapted, multimodality therapy as established in protocols like SIOP-CNS-GCT-96 (5).

The treatment trajectories in our cohort (**Tables 2**, **3**) illustrate a shifting paradigm: while initial management is multimodal, upon multiple recurrences, the therapeutic arsenal narrows predominantly to systemic chemotherapy, as the feasibility of repeat surgery and radiotherapy diminishes due to cumulative toxicity and anatomical constraints (20–24).

### 4.1 Limitations

This study has some limitations. It is a retrospective study, not a randomized trial. It lacks molecular-level and mechanistic investigations (18). The cases included in the study have been treated at multiple hospitals, with various confounding factors. However, this is also an advantage, as it reflects the real-world situation of repeatedly recurrent GCTs. Additionally, the sample size of this study is relatively large, which is another strength. Meanwhile, the absence of a non-recurrent control group and the relatively small sample size limit our ability to detect factors uniquely predictive of multiple relapses. Based on the observed effect size (e.g., HR ≈ 0.6 for chemotherapy vs. radiotherapy), approximately 120–150 patients would be required for 80% power at α = 0.05. Future multicenter prospective studies with matched controls and molecular profiling are therefore warranted to validate these hypothesis-generating findings. Finally, the lack of centralized pathology review and molecular profiling data prevents exploration of biological determinants of aggressive, recurrent disease. A multicenter collaboration with a larger sample size (e.g., >200 patients) would be required to achieve adequate statistical power. Ideally, this should be conducted as a prospective cohort with centralized pathology review and molecular profiling (e.g., methylation subtyping, driver gene analysis) to identify biological subtypes underlying the multiply recurrent phenotype.

## 5 Conclusion

For patients with multiply recurrent GCTs, management increasingly relies on towards systemic chemotherapy as local modalities become less feasible. In addition, the interval between recurrences shortens with successive relapses, with the time to the second recurrence being significantly shorter than that to the first in both germinomas and NGGCTs.

## Data Availability

The original contributions presented in the study are included in the article/supplementary material. Further inquiries can be directed to the corresponding author/s.

## References

[1] Bowzyk Al-Naeeb A Murray M Horan G Harris F Kortmann RD Nicholson J . Current management of intracranial germ cell tumours. Clin Oncol (R Coll Radiol). (2018) 30:204–14. doi: 10.1016/j.clon.2018.01.009 29454505

[2] M M S K T K F T N O F N . Primary intracranial germ cell tumors: a clinical analysis of 153 histologically verified cases. J Neurosurg. (1997) 86:446-55. doi: 10.3171/jns.1997.86.3.0446 9046301

[3] Fetcko K Dey M . Primary central nervous system germ cell tumors: a review and update. Med Res Arch. (2018) 6. doi: 10.18103/mra.v6i3.1719 30271875 PMC6157918

[4] Echevarría ME Fangusaro J Goldman S . Pediatric central nervous system germ cell tumors: a review. Oncologist. (2008) 13:690–9. doi: 10.1634/theoncologist.2008-0037 18586924

[5] Calaminus G Frappaz D Kortmann RD Krefeld B Saran F Pietsch T . Outcome of patients with intracranial non-germinomatous germ cell tumors-lessons from the SIOP-CNS-GCT-96 trial. Neuro Oncol. (2017) 19:1661–72. doi: 10.1093/neuonc/nox122 29048505 PMC5716085

[6] Goodwin TL Sainani K Fisher PG . Incidence patterns of central nervous system germ cell tumors: a SEER study. J Pediatr Hematol Oncol. (2009) 31:541–4. doi: 10.1097/MPH.0b013e3181983af5 19636276

[7] Villano JL Virk IY Ramirez V Propp JM Engelhard HH McCarthy BJ . Descriptive epidemiology of central nervous system germ cell tumors: nonpineal analysis. Neuro Oncol. (2010) 12:257–64. doi: 10.1093/neuonc/nop029 20167813 PMC2940596

[8] Haley G Gino C Toni V-K Quinn TO Carol K Diana SO . Descriptive epidemiology of germ cell tumors of the central nervous system diagnosed in the United States from 2006 to 2015. J Neuro-Oncol. (2019) 143. doi: 10.1007/s11060-019-03173-4 31025275

[9] Murray MJ Bailey S Heinemann K Mann J Göbel UK Saran F . Treatment and outcomes of UK and German patients with relapsed intracranial germ cell tumors following uniform first-line therapy. Int J Cancer. (2017) 141:621–35. doi: 10.1002/ijc.30755 28463397

[10] Hu YW Huang PI Wong TT Ho DM Chang KP Guo WY . Salvage treatment for recurrent intracranial germinoma after reduced-volume radiotherapy: a single-institution experience and review of the literature. Int J Radiat Oncol Biol Phys. (2012) 84:639–47. doi: 10.1016/j.ijrobp.2011.12.052 22361082

[11] Nakamura H Takeshima H Makino K Kochi M Ushio Y Kuratsu J . Recurrent intracranial germinoma outside the initial radiation field: a single-institution study. Acta Oncol. (2006) 45:476–83. doi: 10.1080/02841860500519778 16760185

[12] Seah T Zhang C Halbert J Prabha S Gupta S . The magnitude and predictors of therapy abandonment in pediatric central nervous system tumors in low- and middle-income countries: systematic review and meta-analysis. Pediatr Blood Cancer. (2019) 66:e27692. doi: 10.1002/pbc.27692 30835958

[13] Liang SY Yang TF Chen YW Liang ML Chen HH Chang KP . Neuropsychological functions and quality of life in survived patients with intracranial germ cell tumors after treatment. Neuro Oncol. (2013) 15:1543–51. doi: 10.1093/neuonc/not127 24101738 PMC3813422

[14] Acharya S DeWees T Shinohara ET Perkins SM . Long-term outcomes and late effects for childhood and young adulthood intracranial germinomas. Neuro Oncol. (2015) 17:741–6. doi: 10.1093/neuonc/nou311 25422317 PMC4482856

[15] Kamoshima Y Sawamura Y Ikeda J Shirato H Aoyama H . Late recurrence and salvage therapy of CNS germinomas. J Neuro-Oncol. (2008) 90:205–11. doi: 10.1007/s11060-008-9649-7 18604473

[16] Matthew JM Ute B Ryo N Jason F Masao M James CN . Consensus on the management of intracranial germ-cell tumours. Lancet Oncol. (2015) 16:e470–e477. doi: 10.1016/s1470-2045(15)00244-2 26370356

[17] Fukushima S Yamashita S Kobayashi H Takami H Fukuoka K Nakamura T . Genome-wide methylation profiles in primary intracranial germ cell tumors indicate a primordial germ cell origin for germinomas. Acta Neuropathol. (2017) 133:445–62. doi: 10.1007/s00401-017-1673-2 28078450

[18] Zhou J Wu C Li S . CNS germ cell tumors: molecular advances, significance in risk stratification and future directions. Brain Sci. (2024) 14. doi: 10.3390/brainsci14050445 38790424 PMC11119131

[19] Bo L Shuang Z Shouwei L Chunde L Wei L Lin L . Novel molecular subtypes of intracranial germ cell tumors expand therapeutic opportunities. Neuro Oncol. (2024) 26:1335–51. doi: 10.1093/neuonc/noae038 38430549 PMC11226877

[20] Shirato H Aoyama H Ikeda J Fujieda K Kato N Ishi N . Impact of margin for target volume in low-dose involved field radiotherapy after induction chemotherapy for intracranial germinoma. Int J Radiat Oncol Biol Phys. (2004) 60:214–7. doi: 10.1016/j.ijrobp.2004.02.024 15337558

[21] Calaminus G Kortmann R Worch J Nicholson JC Alapetite C Garrè ML . SIOP CNS GCT 96: final report of outcome of a prospective, multinational nonrandomized trial for children and adults with intracranial germinoma, comparing craniospinal irradiation alone with chemotherapy followed by focal primary site irradiation for patients with localized disease. Neuro Oncol. (2013) 15:788–96. doi: 10.1093/neuonc/not019 23460321 PMC3661100

[22] Alapetite C Brisse H Patte C Raquin MA Gaboriaud G Carrie C . Pattern of relapse and outcome of non-metastatic germinoma patients treated with chemotherapy and limited field radiation: the SFOP experience. Neuro Oncol. (2010) 12:1318–25. doi: 10.1093/neuonc/noq093 20716594 PMC3018943

[23] Balmaceda C Heller G Rosenblum M Diez B Villablanca JG Kellie S . Chemotherapy without irradiation--a novel approach for newly diagnosed CNS germ cell tumors: results of an international cooperative trial. The First International Central Nervous System Germ Cell Tumor Study. J Clin Oncol. (1996) 14:2908–15. doi: 10.1200/jco.1996.14.11.2908 8918487

[24] Perez-Somarriba M Moreno-Tejero ML Rozas MI Pelaez I Madero L Lassaletta A . Gemcitabine, paclitaxel, and oxaliplatin (GEMPOX) in the treatment of relapsed/refractory intracranial nongerminomatous germ cell tumors. Pediatr Blood Cancer. (2020) 67:e28089. doi: 10.1002/pbc.28089 31724795

